# Supplementary material for: Citizen science improves our understanding of the impact of soil management on wild pollinator abundance in agroecosystems
Source: PLoS One. 2020 Mar 10;15(3):e0230007. doi: 10.1371/journal.pone.0230007 (PMC7064200; doi:10.1371/journal.pone.0230007)
Supplement: S1 Fig — Surveys were submitted via a smartphone application, web browser, or mail. (PDF) [file pone.0230007.s001.pdf]

Please contact us if you have any question about the survey
Logan Appenfeller at appenfel@msu.edu or Zsofia Szendrei at szendrei@msu.edu

By filling out this form you are participating in a citizen science research project focusing on squash bees. It was initiated by Michigan State University's Vegetable Entomology Lab. With your help, our goal is to learn about the abundance and distribution of squash bees that specialize on cucurbits and are native to Michigan.
This project is funded by the USDA Organic Program.
You indicate your voluntary agreement to participate by completing and returning this survey. Your participation in this survey is voluntary, and thank you for participating!
Questions marked with an Asterisk are required for survey submission, others are optional.

Name (optional): ____

Contact (email preferred, optional): ____

*What are the last 4 digits of your phone number? ___________________

*What vine crops are grown in the garden? (Check all that apply)

- Summer Squash (Yellow, Zucchini, Pattypan, Crookneck, etc.)
- Winter Squash (Butternut, Acorn, Buttercup, Delicata, Hubbard, Kabocha, Pumpkin, Spaghetti, etc.)
- Cucumber (Salad, Pickling, Slicing, etc.)
- Melon (Cantaloupe, Honeydew, Muskmelon, Watermelon, etc.)

*Do you consider yourself an organic grower? ○ Yes ○ No

*What type of tillage do you use?

- Full Tillage (100% soil cultivation)
- Reduced Tillage (partial soil cultivation)
- No Tillage (no cultivation)

*What is your tillage depth (inches)?

○ 0 ○ 1-5 ○ 6-10 ○ 11-20

*How many types of vegetable crops do you grow? ___________________________________

*What is the total area of your garden/farm?

○ < 1 acre ○ 1 - 5 acre ○ > 1 acre

*How much area do you grow cucurbits on?

○ < 0.5 acre ○ 0.5 - 1 acre ○ > 1 acre

*How do you irrigate your vine crops? (Check all that apply)

○ Trickle/Drip ○ Overhead/Sprinkler/By hand ○ None

*What insecticides do you use on your vine crops? (Check all that apply)

- Approved for organic use, Biopesticides
- Restricted use or conventional synthetic pesticides
- None

*What type of mulch do you use with your cucurbit plants? (Check all that apply)

○ Plastic ○ Plant Material ○ None

RETURNING THE SURVEY: Please EMAIL this survey to Zsofia Szendrei at szendrei@msu.edu
or MAIL to 1129 Farm Lane, Room 348, East Lansing MI 48824

Please contact us if you have any question about the survey
Logan Appenfeller at appenfel@msu.edu or Zsofia Szendrei at szendrei@msu.edu

Bee Observation Protocol

Please observe 5 flowers on your vine crop during peak bloom.
Observe each flower for 1 minute, for a total of 5 minutes.
Add up the number of bees observed during the 5 minute observation and record in the categories below. Please avoid counting the same bee twice.
Bee observations should only be done in the morning of sunny days with no more than light winds, air temperature should be 70°F or above.
You can submit as many observations as you would like. For each observation, please use a new observation form.
Please categorize bees into one of 4 groups: **squash, honey, bumble, 'other'**
To see pictures of bees and get help with identification go to https://goo.gl/Yb0VFS
To watch a video about squash bees vs. honey bees go to https://youtu.be/a2UcgRx9ugE

*Date: ______________________________

*What is the nearest town/city to your current location? (Town/City, State)

____

*What vine crop are you observing?

- ○ Summer Squash (Yellow, Zucchini, Pattypan, Crookneck, etc.)
- ○ Winter Squash (Butternut, Acorn, Buttercup, Delicata, Hubbard, Kabocha, Pumpkin, Spaghetti, etc.)
- ○ Cucumber (Salad, Pickling, Slicing, etc.)
- ○ Melon (Cantaloupe, Honeydew, Muskmelon, Watermelon, etc.)

	# of Squash Bee	# of Honey Bee	# of Bumble Bee	# of Other Bees
Flower # 1				
Flower # 2				
Flower # 3				
Flower # 4				
Flower # 5				

RETURNING THE SURVEY: Please EMAIL this survey to Zsofia Szendrei at szendrei@msu.edu
or MAIL to 1129 Farm Lane, Room 348, East Lansing MI 48824
