# Supplementary material for: Citizen science improves our understanding of the impact of soil management on wild pollinator abundance in agroecosystems
Source: PLoS One. 2020 Mar 10;15(3):e0230007. doi: 10.1371/journal.pone.0230007 (PMC7064200; doi:10.1371/journal.pone.0230007)
Supplement: S1 Table — Number of observations by cucurbit management method, mean ± SEM, and Kruskal-Wallis test results for honey bees, bumble bees, and other bees in a citizen science survey conducted in 2017–2019. Significant pairwise differences among factor levels were determined using Dunn’s test and are indicated by different letters following mean ± SEM values (α < 0.05). (DOCX) [file pone.0230007.s002.docx]

| **S1 Table. Data summary and results of statistical analyses for honey bees, bumble bees, and other bees.** Number of observations by cucurbit management method and mean ± SEM, Kruskal-Wallis test results for honey bees, bumble bees, and other bees in a citizen science survey conducted in 2017-2019. Significant pairwise differences among factor levels were determined using Dunn’s test and are indicated by different letters following mean ± SEM values ($\alpha<0.05$). | | | | | | | | |
| --- | --- | --- | --- | --- | --- | --- | --- | --- |
|  |  | **Tillage type** | | |  |  |  |  |
|  |  | **None** | **Reduced** | **Full** |  | ${}^{\boldsymbol{2}}$ | **df** | ***P* value** |
| **Honey bees** | N | 109 | 97 | 19 |  |  |  |  |
|  | mean ± SEM | 0.29 ± 0.06 | 0.33 ± 0.06 | 0.47 ± 0.18 |  | 1.40 | 2 | 0.50 |
| **Bumble bees** | N | 103 | 101 | 24 |  |  |  |  |
|  | mean ± SEM | 0.43 ± 0.06 | 0.58 ± 0.07 | 0.67 ± 0.17 |  | 2.96 | 2 | 0.23 |
| **Other bees** | N | 110 | 111 | 25 |  |  |  |  |
|  | mean ± SEM | 1.33 ± 0.14a | 0.68 ± 0.09b | 1.36 ± 0.32ab |  | 11.04 | 2 | <0.01 |
|  | | | | | | | | |
|  |  | **Tillage depth (cm)** | | |  |  |  |  |
|  |  | **0** | **3-14** | **15-25** |  | ${}^{\boldsymbol{2}}$ | **df** | ***P* value** |
| **Honey bees** | N | 109 | 96 | 20 |  |  |  |  |
|  | mean ± SEM | 0.29 ± 0.06 | 0.35 ± 0.06 | 0.35 ± 0.13 |  | 1.05 | 2 | 0.59 |
| **Bumble bees** | N | 103 | 102 | 23 |  |  |  |  |
|  | mean ± SEM | 0.43 ± 0.06 | 0.65 ± 0.08 | 0.39 ± 0.14 |  | 5.05 | 2 | 0.08 |
| **Other bees** | N | 110 | 112 | 24 |  |  |  |  |
|  | mean ± SEM | 1.33 ± 0.14a | 0.78 ± 0.10b | 0.96 ± 0.24ab |  | 8.66 | 2 | 0.01 |
|  | | | | | | | | |
|  |  | **Mulch** | | | |  |  |  |
|  |  | **None** | **Plant** | **Plastic** | **Plastic + Plant** | ${}^{\boldsymbol{2}}$ | **df** | ***P* value** |
| **Honey bees** | N | 78 | 124 | 5 | 18 |  |  |  |
|  | mean ± SEM | 0.13 ± 0.04a | 0.44 ± 0.06b | 0.40 ± 0.40ab | 0.33 ± 0.14ab | 12.02 | 3 | <0.01 |
| **Bumble bees** | N | 79 | 119 | 6 | 24 |  |  |  |
|  | mean ± SEM | 0.56 ± 0.08 | 0.53 ± 0.07 | 0.33 ± 0.33 | 0.42 ± 0.13 | 1.56 | 3 | 0.67 |
| **Other bees** | N | 81 | 135 | 6 | 24 |  |  |  |
|  | mean ± SEM | 0.85 ± 0.12 | 1.19 ± 0.12 | 1.83 ± 0.91 | 0.63 ± 0.16 | 4.26 | 3 | 0.23 |
|  | | | | | | | | |
|  |  | **Irrigation** | | |  |  |  |  |
|  |  | **Overhead** | **Drip** | **Overhead + Drip** |  | ${}^{\boldsymbol{2}}$ | **df** | ***P* value** |
| **Honey bees** | N | 178 | 37 | 10 |  |  |  |  |
|  | mean ± SEM | 0.31 ± 0.05 | 0.37 ± 0.11 | 0.30 ± 0.15 |  | 0.30 | 2 | 0.86 |
| **Bumble bees** | N | 176 | 39 | 13 |  |  |  |  |
|  | mean ± SEM | 0.57 ± 0.06 | 0.41 ± 0.11 | 0.23 ± 0.12 |  | 4.13 | 2 | 0.13 |
| **Other bees** | N | 190 | 43 | 13 |  |  |  |  |
|  | mean ± SEM | 1.01 ± 0.09 | 1.21 ± 0.23 | 1.00 ± 0.23 |  | 0.58 | 2 | 0.75 |
|  |  |  | | |  |  |  |  |
|  |  | **Insecticides** | | |  |  |  |  |
|  |  | **None** | **Organic** | **Conventional** |  | ${}^{\boldsymbol{2}}$ | **df** | ***P* value** |
| **Honey bees** | N | 184 | 31 | 10 |  |  |  |  |
|  | mean ± SEM | 0.31 ± 0.04 | 0.42 ± 0.12 | 0.30 ± 0.21 |  | 1.13 | 2 | 0.57 |
| **Bumble bees** | N | 181 | 34 | 13 |  |  |  |  |
|  | mean ± SEM | 0.56 ± 0.06 | 0.29 ± 0.09 | 0.62 ± 0.21 |  | 3.62 | 2 | 0.16 |
| **Other bees** | N | 196 | 35 | 15 |  |  |  |  |
|  | mean ± SEM | 1.14 ± 0.10 | 0.63 ± 0.15 | 0.67 ± 0.23 |  | 4.77 | 2 | 0.09 |
|  |  |  | |  |  |  |  |  |
|  |  | **Cucurbit area (hectare)** | |  |  |  |  |  |
|  |  | **< 0.4** | **> 0.4** |  |  | ${}^{\boldsymbol{2}}$ | **df** | ***P* value** |
| **Honey bees** | N | 214 | 11 |  |  |  |  |  |
|  | mean ± SEM | 0.31 ± 0.04 | 0.64 ± 0.24 |  |  | 2.92 | 1 | 0.09 |
| **Bumble bees** | N | 215 | 13 |  |  |  |  |  |
|  | mean ± SEM | 0.54 ± 0.05 | 0.15 ± 0.10 |  |  | 3.60 | 1 | 0.06 |
| **Other bees** | N | 232 | 14 |  |  |  |  |  |
|  | mean ± SEM | 1.06 ± 0.09 | 0.79 ± 0.19 |  |  | 0.01 | 1 | 0.93 |
|  |  |  | | |  |  |  |  |
|  |  | **Vine crop observed** | | | |  |  |  |
|  |  | **Mixed** | **Summer** | **Summer + Winter** | **Winter** | ${}^{\boldsymbol{2}}$ | **df** | ***P* value** |
| **Honey bees** | N | 35 | 107 | 18 | 65 |  |  |  |
|  | mean ± SEM | 0.29 ± 0.11 | 0.26 ± 0.06 | 0.50 ± 0.17 | 0.40 ± 0.08 | 6.50 | 3 | 0.09 |
| **Bumble bees** | N | 34 | 105 | 20 | 69 |  |  |  |
|  | mean ± SEM | 0.35 ± 0.12a | 0.36 ± 0.06a | 0.70 ± 0.18ab | 0.80 ± 0.09b | 20.56 | 3 | <0.01 |
| **Other bees** | N | 39 | 113 | 20 | 74 |  |  |  |
|  | mean ± SEM | 0.95 ± 0.21 | 1.21 ± 0.13 | 1.10 ± 0.30 | 0.81 ± 0.14 | 6.07 | 3 | 0.11 |
