# Supplementary material for: Citizen science improves our understanding of the impact of soil management on wild pollinator abundance in agroecosystems
Source: PLoS One. 2020 Mar 10;15(3):e0230007. doi: 10.1371/journal.pone.0230007 (PMC7064200; doi:10.1371/journal.pone.0230007)
Supplement: Attachment — Submitted filename: PLOs ONE Review 1.7.20.docx [file pone.0230007.s003.docx]

**Title: Citizen science improves our understanding of native pollinator management**

**Authors: Logan Appenfeller, Zsofia Szendrei, Sarah Lloyd**

The authors of this paper have conducted a well replicated study examining the effects of farm management, especially tillage and practices related to soil, and how these practices effect the abundance of squash bees and other bees visiting cucurbits in Michigan by using citizen science data reported over three summers. Overall, they found a greater total number of squash bees reported than honey bees, bumble bees, and other bees. No till and reduced tillage practices supported greater numbers of squash bees than full till practices and the use of mulch had no effect on squash bee abundance. Tillage and tillage depth did not have an effect on honey bee and bumble bee abundance, however, other bees were significantly affected by these features with greater numbers of other bees in no till and reduced tillage farms compared to full till. The results of this study are pertinent as wild bees are in decline globally. It is essential to understand how farm management affects bees in order to create management practices to support greater bee communities.

**General Comments:** I commend the writer for a well written and easy to read paper. The topic was very interesting and pertinent. The graphs and figures all look very nice. See below for some suggestions to edit.

There was poor organization of the supplemental table making it a bit hard to digest

Inconsistent use of the oxford comma.

The use of a period after figure in the text and captions is inconsistent (e.g., fig 1, fig.1, fig.1.). Please check these throughout.

**Line 39.** The sentence that starts with “Management practices that increase…” you state that practices which increase bee abundance and richness can ameliorate pollinator deficiencies. However, I think I understand that you mean to say that increased pollinator abundance and richness will ameliorate crop pollination deficiencies, especially for crops that are more effectively pollinated by native bees.

**Line 45.** You explain that because native bees are hard to study many researchers are relying on citizen science data to map abundance and distributions of native bees, however, the reference [15] you provide is not for wild bee data. It may be better to use this reference more generally, stating that citizen science data allow researchers to obtain a greater quantity of samples from a larger geographic region, and then focus on more recent and bee related studies to support that this is being done with native bees. See some examples references:

Mason, L., & Arathi, H. (2019). Assessing the efficacy of citizen scientists monitoring native bees in urban areas. Global Ecology and Conservation, 17, E00561.

Lander, T. (2019). Network modelling, citizen science and targeted interventions to predict, monitor and reverse bee decline. Plants People Planet  [**https://doi.org/10.1002/ppp3.10068**](https://doi.org/10.1002/ppp3.10068)

Graham, J., Tan, Q., Jones, L., & Ellis, J. (2014). Native Buzz: Citizen scientists creating nesting habitat for solitary bees and wasps. *Florida Scientist,* *77*(4), 204-218. Retrieved January 7, 2020, from www.jstor.org/stable/24321925

**Line 56.** Italicize the genus *Cucurbita*

**Lines 57-71.** This paragraph focuses on explaining the potential for management practices to disturb squash bees and then outlines how previous research has found mixed results for various types of disturbances. The next paragraph on lines 72-78 discuss that you use citizen science to assess how land management in Michigan impacts squash bees. The link between previous work and why you are doing this study is missing. How will your study bridge the gap in knowledge and or clarify the already mixed results that exists on this topic?

Based on what I am reading I would guess that your reasoning is as follows: Multiple studies have assessed how various farm management practices impact the squash bee. However, many of these studies are finding mixed results, do not investigate more than one management practice in the same study, and often suffer from reduced sample sizes. In this study you attempt to clarify how farm management impacts squash bees by assessing multiple management practices such as soil tillage, depth, and mulch. You use citizen science to conduct this study because it will allow you to increase your sample size, therefore, strengthening the magnitude of your result, as well as educate the public on the importance of squash bees.

**Line 82-83.** In some cases you capitalize Master Gardeners and in others you spell it as Master gardeners. Please pick one and check it throughout.

**Line 161.** Hyphenate “Kruskal Wallis” as it is done in line 152

**Line 217.** For the sake of transparency, can you go ahead and just add the coordinating stat for each of the features listed (e.g., insecticides, irrigation, and vine crop)?

**Paragraph beginning on Line 225.** In your statistical analysis section you stated that you performed Dunn post hoc analysis when the overall Kruskal-Wallis test was significant to determine differences in honey bee, bumble bee, and other bees response to farm management, however, it does not appear that you report post hoc stats for any of these differences. Maybe I am misunderstanding this type of test, but it appears to me that you report the main test result and then you are simply stating differences based on the mean. I also do not see the post hoc differences listed in SI Table 1. Can you add the appropriate stats?

**For the Discussion.**

Seeing that you have taken the time to describe how mixed the results are in literature about how farm management affects squash bees, you may consider comparing the results of your study to these previous claims in addition to explaining the reasoning behind your results. I will leave this up to the authors.

If you are not over the word limit you may want to discuss the findings of the honey bee, bumble bee, and other bee data a bit more. As it stands now you have only 1 sentence (line 300) about this. It is not super clear to me what the benefit of measuring these farm practices was for other bees in reference to your study and how it relates to the squash bees. I think I know what you were going for but it is not clearly described. As it is now, it just seems like extra data that got tossed in and were never discussed. Given the differences in nesting and dietary habits, I think you may have more to elaborate on.

**Line 299.** The jump from geographic range to flower visitation in the next sentence confuses me. These two things are not the same. Could you discuss the geographic expansion compared to historical records? Are there any ideas as to why these expansions happened? Is it likely a product of the lack of people reporting or is it because of an increase in farming for Cucurbits by small farmers? My guess is that it is related to the lack of studies and reports by farmers, again highlighting the usefulness of this type of citizen science.
